# Psychosocial Predictors of Psychological Well-Being During the COVID-19 Pandemic

**DOI:** 10.1093/geroni/igab046.3728

**Published:** 2021-12-17

**Authors:** Yeon Ji Ryou, Gina Lee, Rotem Arieli, Peter Martin, Shinae Choi, Jinmyoung Cho, Melinda Heinz

**Affiliations:** 1 Iowa State University, Ames, Iowa, United States; 2 The University of Alabama, Tuscaloosa, Alabama, United States; 3 Baylor Scott & White Health Research Institute, Temple, Texas, United States; 4 Upper Iowa University, Fayette, Iowa, United States

## Abstract

People worldwide have been largely affected by the COVID-19 outbreak. In addition to worries about physical health, it also causes concerns about psychological and mental health. This research aims to explore predictors affecting psychological well-being during the pandemic using the 2018 Health and Retirement Study (HRS) RAND longitudinal data (N = 42,233) and the 2020 HRS COVID-19 module (N = 3,266). Demographics (i.e., gender, age, and education), psychosocial (i.e., personality traits), and health (i.e., comorbidity) variables were included in multivariate logistic and ordinary least square regression analyses predicting feeling “overwhelmed,” “stressed,” and “lonely” during the pandemic. Our results indicated that neuroticism was positively associated with all outcomes. Women were more likely to feel overwhelmed, stressed, and lonely compared to men. Age negatively predicted the overwhelmed and stressed feelings. Furthermore, the effect of depressive symptoms in 2012, 2014, 2016, and 2018 on psychological well-being was assessed by conducting a latent growth curve model. Findings indicate that initial level and increasing change of depressive symptoms over four-time points (waves 11-14) were positively related to psychological feelings. A higher level of depressive symptoms at the initial level of 2012 and increasing reports of depression symptoms predicted higher rates of being stressed, feeling overwhelmed, and lonely during the COVID-19 pandemic. The results have implications for future research and interventions that should target the emotional antecedents and consequences of pandemics.

